# Two novel pathogenic variants in the *TCOF1* found in two Chinese cases of Treacher Collins syndrome

**DOI:** 10.1002/mgg3.2405

**Published:** 2024-03-05

**Authors:** Dan‐Yan Zhuang, Shu‐Ni Sun, Zhuo‐Jie Hu, Min Xie, Yu‐Xin Zhang, Lu‐Lu Yan, Jie‐Wen Pan, Hai‐bo Li

**Affiliations:** ^1^ The Central Laboratory of Birth Defects Prevention and Control Women and Children's Hospital of Ningbo University Ningbo Zhejiang China; ^2^ Department of Neonatology Women and Children’ s Hospital of Ningbo University Ningbo Zhejiang China; ^3^ Department of Child Health Care Women and Children's Hospital of Ningbo University Ningbo Zhejiang China

**Keywords:** craniofacial dysplasia, novel variation, *TCOF1*, Treacher Collins syndrome

## Abstract

**Background:**

Treacher Collins Ι syndrome (TCS1, OMIM:154500) is an autosomal dominant disease with a series of clinical manifestations such as craniofacial dysplasia including eye and ear abnormalities, small jaw deformity, cleft lip, as well as repeated respiratory tract infection and conductive hearing loss. Two cases of Treacher Collins syndrome with *TCOF1*(OMIM:606847) gene variations were reported in the article, with clinical characteristics, gene variants and the etiology.

**Methods:**

The clinical data of two patients with Treacher Collins syndrome caused by *TCOF1* gene variation were retrospectively analyzed. The whole exome sequencing (WES) was performed to detect the pathogenic variants of *TCOF1* gene in the patients, and the verification of variants were confirmed by Sanger sequencing.

**Results:**

Proband 1 presented with bilateral craniofacial deformities, conductive hearing loss and recurrent respiratory tract infection. Proband 2 showed bilateral craniofacial malformations with cleft palate, which harbored similar manifestations in her family. She died soon after birth due to dyspnea and feeding difficulties. WES identified two novel pathogenic variants of *TCOF1* gene in two probands, each with one variant. According to the American College of Medical Genetics and Genomics, the heterozygous variation NM_001371623.1: c.877del (p. Ala293Profs*34) of *TCOF1* gene was detected in Proband 1, which was evaluated as a likely pathogenic (LP) and de novo variant. Another variant found in Proband 2 was NM_001135243.1: c.1660_1661del (p. D554Qfs*3) heterozygous variation, which was evaluated as a pathogenic variation and the variant inherited from the mother. To date, the two variants have not been reported before.

**Conclusion:**

Our study found two novel pathogenic variants of *TCOF1* gene and clarified the etiology of Treacher Collins syndrome. We also enriched the phenotypic spectrum of Treacher Collins syndrome and *TCOF1* gene variation spectrum in the Chinese population, and provided the basis for clinical diagnosis, treatment and genetic counseling.

## Graphical abstract

Our study identified two novel pathogenic variants of TCOF1 gene, clarified the etiology of Treacher Collins syndrome, enriched the phenotypic spectrum of Treacher Collins syndrome and TCOF1 gene variation spectrum in the Chinese population, and provided the basis for clinical diagnosis, treatment and genetic counseling.

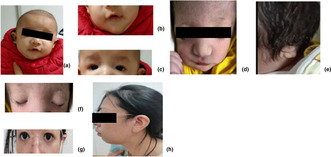

## 1 INTRODUCTION

Treacher Collins Ι syndrome (TCS1, OMIM:154500), also known as bird face syndrome, was an autosomal dominant disease. The incidence of the disease was estimated to be about 1/50,000 (Tse, 2016). The typical manifestations of this disease are craniofacial dysplasia, including the eye abnormalities (cleft palate, eyelid defects, partial loss of lower eyelashes, etc.), ear abnormalities (preauricular fistula, auricle malformation, external auditory canal atresia, external auditory canal/middle ear canal loss, inverted ear, low ear position, etc.), malar arch and mandibular hypoplasia, largemouth and small jaw deformity, cleft lip and palate and velopharyngeal insufficiency, as well as repeated respiratory tract infection, conductive hearing loss (abnormal development of auditory ossicles), etc. (Walker‐Kopp et al., 2017). To date, only four pathogenic genes (*TCOF1* gene, *POLR1B* gene, *POLR1D* gene and *POLR1C* gene) have been identified as the cause of 90% of TCS patients (Fan et al., 2019; Pan et al., 2021), most of these patients are caused by *TCOF1* gene variation. *TCOF1* variants were associated with the clinical phenotype of TCS patients. Patients with *TCOF1* pathogenic variants showed typical phenotypes, namely mandibular hypoplasia (82.8%), zygomatic hypoplasia (83.3%) and lower oblique palpebral fissure (88.4%). Their analysis also showed that 16.2% of patients had speech and/or psychomotor problems. In addition, the clinical characteristics of the *TCOF1* variant are correlated with different exon positions, races and variant types. It is concluded that patients with the 15th exon variant have a significantly reduced incidence of microtia (23.1%), conductive deafness (7.7%) and external auditory canal atresia (0%). Patients with the deficiency exhibit a higher frequency of cleft palate (44%) and intellectual disability (22.2%) (Ulhaq et al., 2023).We analyzed the genetic variation of two TCS1 patients in Ningbo Women and Children's Hospital, and verified the family variation sites and identified the origin of variation by Sanger sequencing, which provided the basis for subsequent clinical diagnosis and treatment and fertility guidance.

## 2 OBJECTS AND METHODS

### 2.1 Ethical compliance

This study was approved by the ethics committee of Ningbo women and children's Hospital (EC2020‐048), both parents of the subjects signed informed consent.

### 2.2 Patients and clinical data

**FIGURE 1:**
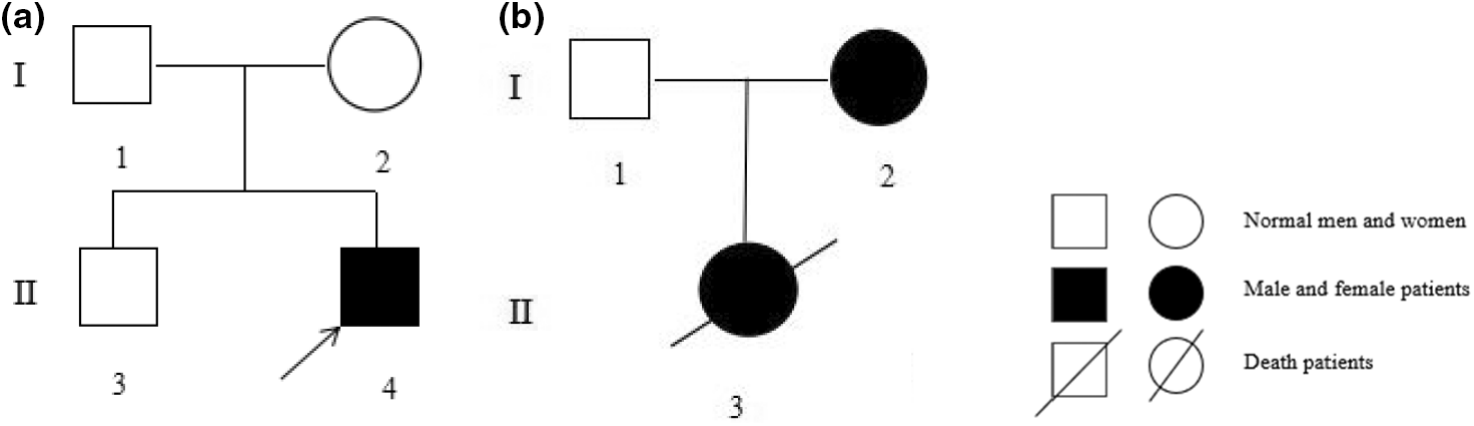
(a) Genetic pedigree of proband 1; (b) Genetic pedigree of proband 2.

**FIGURE 2:**
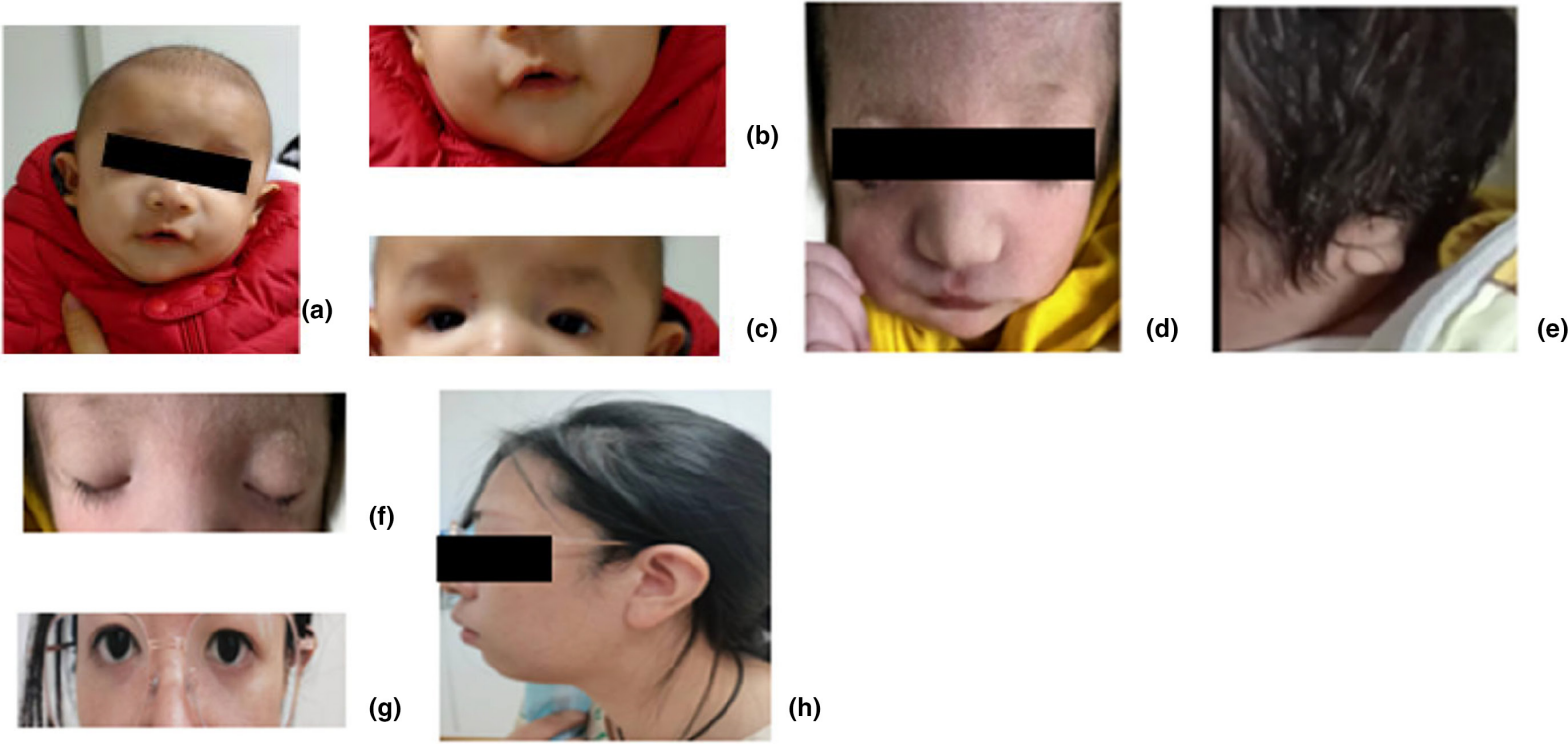
Proband 1: (a, b) small jaw (c) Cleft eye, lower eyelid defect and partial loss of lower eyelashes. proband 2: (d) micrognathia (e) external auditory canal atresia (f) malar hypoplasia, hypoclination of eye fissure, defect of lower eyelid, partial loss of lower eyelash. Mother of Proband 2: (g) eye fissure oblique (h) micromandible.

Proband 1: male, 2 months old, she visited the Department of Pediatrics of Ningbo Women and Children's Hospital on 11 November 2021, due to “abnormal face.” Personal history: G3P2, gestational age of 37^+6^ weeks, natural delivery, birth weight of 2600 g, body length of 49 cm, head circumference of 49 cm, no history of asphyxia, newborn hearing screening and brainstem auditory evoked potential failed, and no abnormality was found in tandem mass spectrometry. Maternal history: fetal three‐dimensional ultrasound results showed no abnormality. Family history: no similar family history, parents health, deny close relatives married (Figure 1a, Genetic pedigree chart). Physical examination: lower eyelid defect, lower ciliary loss, micrognathia (Figure 2a–c); conductive hearing loss, recurrent respiratory tract infection.

The proband 2, a 2‐day‐old child, was admitted to the pediatric clinic of Ningbo Women and Children's Hospital on 10 February 2022. She had died now. G1P1, the gestational age is 37^+2^ Weeks, natural delivery, birth weight of 2830 g, body length of 45 cm, apgar score 10 points, no history of asphyxia, including wide distance between eyes, downward inclination of eye fissure, lower eyelid defect, partial loss of lower eyelashes, micrognathia, malar hypoplasia, atresia of external auditory canal (Figure 2d–f), cleft palate, short limbs, the child died of dyspnea and feeding difficulties during perinatal period. Family history: the mother had similar micrognathia and cleft eyes (Figure 2g,h), her grandfather had a cleft palate and her grandmother had micrognathia. Father in good health, denied consanguineous marriage (Figure 1b, Genetic pedigree chart).

### 2.3 Methods

#### 2.3.1 WES and bioinformatics analysis

In this study, 5 mL peripheral blood of children and their parents were collected and DNA was extracted. Exon capture technology was used. According to the instructions of Illumina sequencing library kit, the library was online. After quantification by the kit, the exons and flanking intron regions of the gene were sequenced and analyzed by Novaseq sequencing platform (Illumina Company, USA). The raw data obtained from sequencing were compared with the reference genome GRCh38/hg38 or UCSC hg19 by BWA software, and GATK and VarScan software were selected to identify and annotate the variants, while data such as sequencing quality, sequencing depth and coverage were counted. Databases such as 1000 Genomes, dbSNP, ExAC, gnomAD, HGMD, OMIM and Clinvar were used to identify suspected pathogenic variants in combination with the clinical phenotype of the children. The pathogenicity of the variants is judged according to the American College of Medical Genetics and Genomics and Genomics and the relevant ClinGen Specialty Group.

#### 2.3.2 Sanger sequencing

Primer 5 software was used to design primers for the screening of suspicious mutations, and the target fragment was amplified and sent to Shanghai Biological Engineering Limited Corporation. The sequencing results were analyzed by SeqMan Pro 8. 0. 2 software. Family co‐segregation analysis was performed by combining the clinical phenotypes of the children and the results of parental validation.

## 3 RESULTS

**FIGURE 3:**
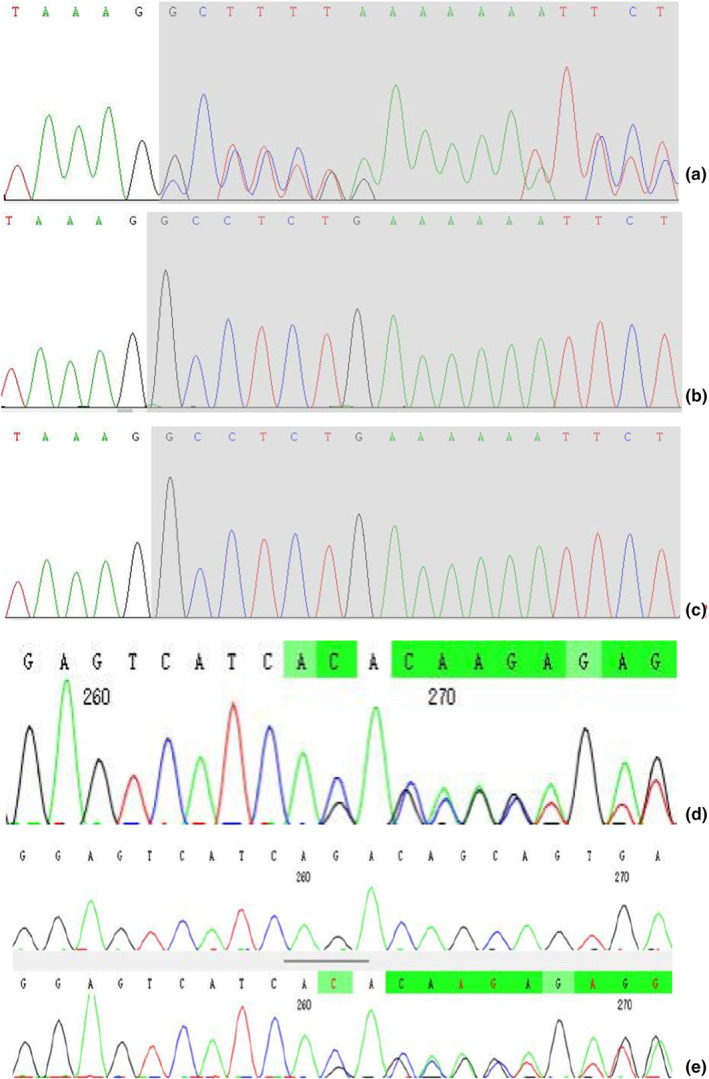
(a) Gene sequencing map: Proband 1 carried the heterozygous variant c.877del: p.Ala293profs*34 in *TCOF1* gene. (b, c) Neither father nor mother of proband 1 carried the c.877del:p.Ala293profs*34 heterozygous variant. (d) Proband 2 carried the variant *TCOF1* gene c.1660_1661del: p.D554Qfs*3 heterozygous variant. (e) The mother of proband 2 carried the *TCOF1* gene c.1660_1661del:p.D554Qfs*3 heterozygous variant, and his father did not carry the variant.

A heterozygous variation NM_001371623.1: c.877del (p. Ala293Profs*34) in the *TCOF1* gene was detected in proband 1 (Figure 3a), and located at Exon11, this variant was not detected in his parents, suggesting that it was a de novo variant (PVS1 + PM2_Supporting), and it was a new variant that had not been reported (Figure 3b,c). The *TCOF1* gene with NM_001135243.1: c.1660_1661del (p. D554Qfs*3) heterozygous variant was detected in Proband 2 (Figure 3d) and confirmed it was inherited from her mother, the mutation was located in Exon8, which was not detected in her father, and was a new pathogenic mutation (PVS1 + PM2_Supporting + PP4) too (Figure 3e).

## 4 DISCUSSION

TCS1 is a rare autosomal dominant disorder defined as congenital craniofacial dysplasia, characterized by unique “bird‐shaped” facial manifestations (malar and mandibular dysplasia, etc.) and conductive hearing loss (auricle, external auditory canal and middle ear malformations) (Pan et al., 2021). The main cause of TCS1 is the variants in *TCOF1* locus, which is located on chromosome 5q32‐q33.1, containing 28 exons and encoding 4465 base pairs (Zhang et al., 2013); It also encodes a low‐complexity, and serine/alanine‐rich protein called the Treacle protein, which is composed of 1488 amino acids with a molecular weight of about 152 KD, and has three domains, namely the N‐terminal and C‐terminal, and a central repetitive domain (Pan et al., 2021). Treacle proteins participate in ribosomal DNA gene transcription by interacting with upstream binding factor (UBF), which is essential for the survival of neural crest cells and plays an important role in craniofacial development during embryonic development (Sakai & Trainor, 2016). As a result, once the variation of *TCOF1* gene occurs, it will lead to insufficient ribosome synthesis and accelerated apoptosis of neuroepithelial cells. The final result is a deficiency of neural crest cells involved in the development of facial bones and cartilage, resulting in craniofacial deformities (Giabicani et al., 2017).The majority of *TCOF1* gene variants are located in exons (Yan et al., 2018). Researchers analyzed TCS1 patients with *TCOF1* variation in different populations, the results showed that exon 10, exon 15, exon 16, exon 23 and exon 24 were the hotspots of gene variation (Splendore et al., 2002).

**FIGURE 4:**
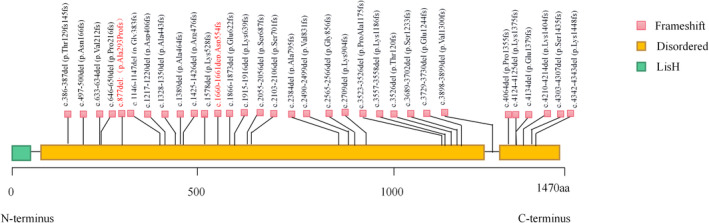
Distribution of 30 possible pathogenic and pathogenic mutation sites in *TCOF1* gene, red was found in this study.

Pan et al. (2021) showed that the hot spots of variants related to TCS1 in the Chinese population were located in exon 5, exon 23 and exon 24, which were different from the hot spot variants of *TCOF1* gene in the two children in this study. According to the human genome mutation database (HGMD), more than 367 sites of variants have been reported in the *TCOF1* gene. The types of *TCOF1* gene variants include the missense variants, the nonsense variants, and the deletions and duplications (So et al., 2004). The most common variants of *TCOF1* gene were deletions and duplications, respectively, accounting for 60% and 25% (Chou et al., 2022; Yan et al., 2018), all of them were frameshift variants. We counted all the deletion variants which had already been reported, as shown in Figure 4. Both deletion variants found in this study were consistent with the above conclusion.

In this study, two cases of *TCOF1* gene variants were detected by WES, one of which was a new variant and the other was inherited from the mother. It has been reported (Wu et al., 2022) that approximately 60% of patients are caused by de novo variants with no family history of the disease. The c.877del (p. Ala293Profs*34) variation of *TCOF1* gene was found in proband 1, which was a heterozygous frameshift deletion variation. The variation is a deletion of one base at position 877 of cDNA, resulting in a change of codon 293 from alanine to proline, and terminate at codon 34, eventually leading to termination of protein translation. The results of Sanger sequencing pedigree validation showed that the parents of proband 1 did not have the variation. Thus, the c.877del (p. Ala293Profs*34) variant was de novo and unreported. The c.1660_1661del (p. D554Qfs*3) variation of *TCOF1* gene was found in proband 2, which was a heterozygous frameshift deletion mutation. This variation was a base deletion at positions 1660 to 1661 of the cDNA, resulting in a change in codon 554 from encoding aspartic acid to glutamine. Then, a stop codon was generated in advance, resulting in the termination of protein translation. The results of family verification by Sanger sequencing showed that the variation of proband 2 was inherited from the mother, and the variation had never been reported. Mother of Proband 2 had a similar presentation (small lower jaw, downward sloping eye fissure) but did not show any other presentation like that of Predecessor 2, with mild symptoms. Her grandfather had cleft palate and her grandmother had a small mandible, which required later genetic testing and family verification.

The symptoms of the two children in this study were consistent with most of those reported in the literature (Nie et al., 2021; Pan et al., 2021; Serrano et al., 2019), they all had typical bilateral craniofacial deformities, such as oblique palpebral fissure, lower eyelid defect, partial loss of lower eyelashes, micrognathia and auricle deformity. The different manifestations of the two cases were that the three‐dimensional ultrasound of the proband 2 showed abnormality with mandible and auricle, which was consistent with the results of prenatal three‐dimensional ultrasound of children with *TCOF1* gene mutation reported by Yan et al. (2018). Besides, Proband 2 was born with a cleft palate, short limbs and a family history of similar presentations, which ended in death due to “breathing difficulties and feeding difficulties.” On the one hand, the cause of Proband 2's death was related to the airway stenosis associated with craniofacial deformity (cleft palate, small mandibular deformity, etc.), which was consistent with the cause of TCS1 death in a newborn reported by Deng et al. (2015). Several researchers (Dixon et al., 1991; Splendore et al., 2000) also confirmed that the airway stenosis and infection associated with craniofacial deformity could cause respiratory failure, resulting in perinatal death.

On the other hand, Proband 2 had feeding difficulties after birth, which was similar to that of a TCS child with *TCOF1* gene variation reported by Giabicani et al. (2017), who had feeding difficulties and digestive intolerance after birth. Presently, this child is dependent on total parenteral nutrition and has not died. But proband 1 does not have feeding difficulties after birth, which needs to be observed. However, proband 1 had no cleft lip and palate and feeding difficulties except craniofacial malformation and repeated respiratory tract infection, and the symptoms were lighter than that of proband 2. Proband 1 also has conductive hearing loss. It has been reported (Kantaputra et al., 2020) that about 50% of TCS patients have different degrees of bilateral conductive hearing loss, which is related to changes in the internal structure of the ear caused by *TCOF1* gene variation.

Richter et al. (2010) showed that the cause of conductive hearing loss caused by *TCOF1* gene variation was the loss of neural crest cells, which led to the abnormal development of auditory bullae (neural crest cell‐derived structures and enveloping the middle ear), and ultimately had a harmful effect on hearing. The presence or absence of this manifestation could not be ascertained as Proband 2 died in the perinatal period.

Otherwise, patients with TCS may also have other manifestations, and the two children in this study did not show. Obstructive sleep apnea can occur in patients with TCS, with an overall prevalence of 46% (54% in children and 41% in adults) (Akre et al., 2012; Plomp et al., 2012), in this study, the proband 1 had no such performance and needed long‐term follow‐up. Moreover, patients with TCS can develop intestinal motility disorders. Barlow et al. (2013) demonstrated the association between the *TCOF1* gene and intestinal dysmotility through a mouse model. They found that mice with the presence of complex Pax3/Tcof1 exhibited a severe reduction in neural crest cell population and migration of neural crest cells to the colon, resulting in intestinal motility disorders in mice. At present, proband 1 in this study does not have such a situation, which requires long‐term follow‐up.

One of the two children reported in this study had died, and the other one was young. In addition to typical craniofacial malformations and repeated respiratory tract infections, some symptoms may not be manifested, and long‐term follow‐up in the outpatient department is required. Timely intervention and treatment are required after other symptoms appear. There is no definitive treatment for TCS. Patients with TCS usually require multidisciplinary (otorhinolaryngology, plastic surgery) treatment, including hearing interventions, jaw plasty, respiratory tract dissection and other symptomatic treatments.

To sum up, if images were found by three‐dimensional ultrasound in fetal period indicates auricle malformation and small mandible, bilateral craniofacial malformation, conductive hearing impairment and other manifestations are found after birth, or there was a similar history in the family, it was necessary to be highly vigilant against TCS. In this paper, two cases of *TCOF1* gene variation were found by WES. That is the first report at home and abroad, which enriches the TCS1 phenotype spectrum and *TCOF1* gene mutation spectrum, and is of great significance for subsequent genetic counseling, disease treatment and fertility guidance.

## AUTHOR CONTRIBUTIONS

All authors have materially participated in the study and manuscript preparation. Dan‐Yan Zhuang and Shu‐Ni Sun carried out all the molecular genetic analysis, and participated in the design of the work; Zhuo‐Jie Hu, Min Xie, Yu‐Xin Zhang, Lu‐Lu Yan and Jie‐Wen Pan collected all clinical data and participated in conceiving the work; Hai‐bo Li designed the work, drafted and revised the manuscript. All authors have approved the final article.

## FUNDING INFORMATION

Municipal Public Welfare Project (2022S035), Ningbo Medical Key Supporting Discipline (2022‐F26), Medical and Health Project of Zhejiang (2023KY1121), Ningbo Top Medical and Health Research Program (2022020405), Ningbo science and technology project (2023Z178 )

## CONFLICT OF INTEREST STATEMENT

The authors declare that the research was conducted in the absence of any commercial or financial relationships that could be construed as a potential conflict of interest.

## Data Availability

The data supporting the conclusions of this article are included within the article and its Supplementary Materials. Further data of this study are available from the corresponding author upon request.
